# The Role of Internet Use and Offline Social Engagement in the Health of Chinese Older Adults: Evidence from Nationally Representative Samples

**DOI:** 10.3390/healthcare11050653

**Published:** 2023-02-23

**Authors:** Chanyan Li, Wanli Mo, Qingwang Wei

**Affiliations:** Department of Psychology, Renmin University of China, Beijing 100872, China

**Keywords:** depression, aging, social engagement, social activities, self-reported health

## Abstract

This study investigated the association between internet use as a novel type of social engagement and the health of older adults, and evaluated the role of online versus offline social activities through nationally representative samples. Participants aged ≥ 60 in the datasets from the Chinese sample of the World Value Survey (N_Sample 1_ = 598) and the China Health and Retirement Longitudinal Study (CHARLS, N_Sample 2_ = 9434) were selected. Correlation analysis presented the positive relationships between internet use and self-reported health in both Sample 1 (*r* = 0.17, *p* < 0.001) and Sample 2 (*r* = 0.09, *p* < 0.001). In addition, the relationships between internet use and both self-reported health and depression (*r* = −0.14, *p* < 0.001) were stronger than the correlation between offline social activities and health outcomes in Sample 2. After accounting for the frequency of traditional social activities, regression analysis revealed the relationship between internet use and higher self-reported health (β_Sample 1_ = 0.16, *p* < 0.001; β_Sample 2_ = 0.04, *p* < 0.001) and lower depressive symptom scores (β = −0.05, *p* < 0.001) This research contributes to the existing body of literature on the favorable relationship between social engagement and health outcomes among Chinese older adults. Additionally, it identifies the social benefits of internet use for health promotion among older adults.

## 1. Introduction

According to the World Health Organization (WHO), approximately one in six people in the world will be 60 or older by 2030, meaning all countries face major challenges from this demographic shift [1], and China is no exception. According to Chinese statistics [2], the population over 60 was 280.04.56 million by the end of 2022, 19.8% of the national total population, and an increase of 0.9% over 2021, indicating that the degree of aging in China is rising. The aging process is not only associated with the deterioration of physical functions, but also with the risk of problems with cognitive, emotional, social, and other psychological functions [3]. Over 180 million Chinese people aged 60 or older suffer from chronic diseases [4], and approximately 40% of older adults aged ≥ 60 years suffer from depressive symptoms [5], indicating changes in the health status of Chinese older adults. Hence, it is vital to pay greater attention to older adults as well as to explore factors that contribute to improving their health status.

According to the WHO’s “active aging: a policy framework” [6], social engagement is important for older adults. Social engagement is the involvement and/or engagement of individuals in social activities, both formal and informal [7,8], including contact with family members, connections with friends and neighbors, involvement in volunteer activities, and participation in local leisure activities [9,10,11]. Extensive research has investigated the broad impacts of social engagement on older people’s health. Higher levels of social engagement are linked to health and well-being as people age [12,13] and have even been shown to protect cognitive function [14,15,16,17].

Although prior studies demonstrated the positive effects of social engagement based on actual interactions, the global coronavirus disease 2019 (COVID-19) pandemic has altered people’s lifestyles, with the sudden limitation of in-person interactions contributing to increased loneliness and social isolation, which are connected to depression and all-cause mortality [18,19]. Another cross-sectional study suggested that subjective social isolation, rather than objective social isolation, was related to psychological distress and depressive symptoms in older adults over 55 [20], suggesting that what provides individuals with involvement and perceived integration in their social networks can mitigate the detrimental impacts of physical separation. The internet might act as this buffer because its usage has not been affected by the epidemic, and older adults can still chat with friends or engage in other social activities online. A longitudinal study using data from the Health and Retirement Study showed a strong correlation between subjective health status and internet use among older adults, and that this relationship was stable between 2008 and 2016 [21]. Moreover, a recent study in China indicated a general decline in social leisure activities and an increase in home-bound and solitary leisure activities [13], indicating that using the internet to meet social demands might be a rising trend for older adults. Additionally, a 9-year longitudinal study found that computer use longitudinally predicted better self-reported physical and mental health and reduced functional disabilities, and that it also helped older adults improve social interactions and have closer friends [22]. There is also evidence that older persons’ health literacy required to manage health can be maintained by internet use and social engagement, and beneficial associations also appear to exist between internet use and cultural engagement [23], showing that internet use has a positive effect similar to traditionally objective social engagement during aging.

To date, few studies have focused on this aspect or considered internet use as social engagement. Researchers prefer to treat internet use as a channel to gain health information or as a health management platform for older adults. Jin et al. [24] emphasized that having access to the internet narrowed the gap between urban and rural hypertension management outcomes, and internet websites that provide people with mental health knowledge play a positive role in mental health literacy [25], while internet-based treatments for people with depressive symptoms have proven effective [26].

However, findings from previous studies offer inconsistent results, indicating that the internet is a double-edged sword for older adults’ health. The results of a cross-sectional survey showed that people over 60 in Switzerland and Poland had problem internet use (PIU), which is defined as the unrestrained use of the internet to satisfy certain needs such emotional coping and improving a sense of belonging and self-esteem [27]. A review also found three studies including populations aged over 55 showing the presence of PIU, even though data were not specifically presented for senior participants [28]. In addition, researchers have emphasized that internet use has an association with declining contact with family, narrowed social networking, and increased loneliness and depression [29]. Other negative outcomes, such as a sedentary lifestyle, psychiatric disorders, and neurological disorders (e.g., depression), cannot be ignored [28].

As mentioned above, the stress of rapid population aging and pandemics, as well as the contradictory results above, underscore the need for more research into the association between internet use and the health of older adults. Furthermore, it is essential to compare the effects of internet use with traditional objective social engagement. Previous studies have confirmed the benefit of traditional social activities, divided into three types—entertainment, self-improvement, and prosocial behavior—according to activity purpose.

Of these types, entertainment activities for personal enjoyment have been the most commonly examined. Bennett’s longitudinal study [30] revealed that older people who had survived at follow-up had a high level of participation in entertainment, reflected in both actual (e.g., attending religious services) and symbolic participation (e.g., reading newspapers). A series of studies discovered that older people with a healthier status have higher levels of entertainment activities, such as playing Ma-jong, cards, or other games [31,32,33].

Self-improvement, reflecting the pursuit of better physical function or individual capability through physical or cognitive stimulation, is another hot topic being studied. Of the leisure activities, physical activity had the strongest association with the survival of people aged 75 or older [34] and was linked to a reduced incidence of dementia [35,36]. Similarly, a review concluded that people engaged in cognitively stimulating activities (e.g., reading or learning during leisure time) also have a lower risk of dementia [15].

Prosocial behavior is another area attracting increasing attention. Most existing studies have reported its positive effect on perceived health [37], with reduced mortality among older adults [38,39], as well as fewer depressive symptoms and lower utilization of healthcare services [40]. A meta-analysis of 16 studies conducted in the United States revealed that participation in volunteer work in later life predicted improved functioning and lower mortality [41].

In summary, prior research has indicated that traditional social engagement activities, including entertainment, self-improvement, and prosocial behavior, play a crucial role in enhancing older adults’ health. Nevertheless, the pandemic has made it difficult for older people to partake in social engagement. Thus, new social participation activities must be found. Due to the fast growth of network technology, the internet has permeated even into the daily routines of older adults. Although numerous studies have examined the link between social engagement and health outcomes of older adults, the important social value of internet use has received little attention, let alone any comparison between traditional and new types of social engagement. Moreover, the majority of previous research concentrated on populations in the United States or Europe, and the data resources used in studies conducted in China lacked timeliness to a certain extent, as many were collected before 2012. Hence, further study is required to explore social engagement patterns among older people in mainland China.

The present work aims to assess the relationship between internet use and health outcomes, including depression and self-reported health among older people, and to compare it with the influence of traditional social activities. We hypothesized that internet use positively predicted self-reported health and negatively predicted depression. We selected Chinese older adults aged ≥ 60 from two nationally representative databases separately to test the hypothesis. Sample 1 was from the dataset of the Chinese older adults from the 7th wave of the World Values Survey (WVS), and Sample 2 was from the dataset of the China Health and Retirement Longitudinal Study (CHARLS). Both Sample 1 and Sample 2 explored the relationship between internet use and health outcomes among older people, and Sample 2 further compared the impact of online and offline social activities.

## 2. Materials and Methods

### 2.1. Data and Participants

Both samples were from nationally representative surveys in China. Sample 1 used the WVS dataset of Chinese older adults to investigate the changing values of the public using stratified multi-stage probability proportional to size sampling (PPS). Specifically, all county-level units were sorted by administrative stratification and the level of internationalization, urbanization, and economic development. Using PPS, 60 countries and districts were selected, for each of which 2 townships or street offices were selected. The WVS is the largest cross-national academic survey conducted in almost 100 countries and helps study subjective well-being [42]. The Chinese sample of wave 7 (2018) of the WVS contained 3036 respondents. Sample 1 in our study included 598 participants aged ≥ 60. Participants were excluded if they aged < 60 (N = 2428) and were listwise deleted if they were missing the variables (N = 10) required for data analysis.

CHARLS, which began in 2011–2012, is a biennial nationally representative observational study that focuses on Chinese middle-aged and elderly people aged ≥ 45. The CHARLS study adopts a stratified (by per capita gross domestic product (GDP)), multi-stage (county/district—village/community—household) PPS sampling method to randomly collect participants. County-level units were stratified by urban district or rural county, in addition to province and GDP per capita, and 150 units of which were selected with PPS. Thus, CHARLS is representative of both rural and urban areas within China. Similarly, three villages and communities were chosen for each urban district and rural country [43]. Therefore, the 2018 round of CHARLS included 19,816 individuals from 450 counties in 28 provinces. Within the scope of this study were evaluations of the participants’ social, economic, and health situations [43]. As our research focused on the influence of social engagement on older adults’ health, we first excluded participants not responding to sections with required variables (N = 99). Participants younger than 60 (n = 6607), as well as those with missing values in the demographic information (N = 69) and investigated variables (N = 3607) were also excluded before analysis. Thus, there were a total of 9434 individuals in Sample 2.

### 2.2. Measures

#### 2.2.1. Dependent Variables

In WVS, participants were asked to rate their state of health on a 5-point Likert rating scale which ranged from 1 = “very poor” to 5 = “very good”, with higher values reflecting a healthier status.

In CHARLS, the measurement of self-reported health was the same as in WVS. In addition, depression was also analyzed as an outcome variable. Depression was measured by a short version of the Center for Epidemiologic Studies Depression (CES-D 10), related to depressed affect, psychomotor retardation, and positive affect [44]. Participants answered how they had been feeling and acting over the past week on a 7-point scale ranging from 1 = “<1 day” to 4 = “5–7 days”. The overall score of the 10 items, after reversing two positive affect items, ranged from 10 to 40, with higher values reflecting greater depression. In this study, the Cronbach alpha for CES-D 10 was 0.74.

#### 2.2.2. Independent Variables

In WVS, given that not everyone had access to all online activities, we chose the highest frequency of all activities rather than the average frequency as the measurement indicator. Thus, internet use was measured by selecting the highest frequency of using a mobile phone, email, computer internet, or social media to obtain information (1 = “never” to 5 = “daily”). There was no item measuring social activity in the WVS, so offline social activities were not able to be analyzed in Sample 1.

In CHARLS, participants reported whether they had taken part in any of the common social activities in the previous month, including internet use and other offline activities. If they reported no participation in these activities, they were scored as 0. If they reported that they participated in these activities, the frequency of involvement in each activity was further counted (1 = “not regularly” to 3 = “almost daily”). These responses were gathered to create a categorical variable ranging from 0 to 3, with higher scores suggesting more social engagement [16,45]. Internet use was the only single-index activity online, with higher scores indicating more internet usage. We excluded stock investment because economic activity involves little social interaction. Next, we divided offline activities into three categories according to their content: (i) entertainment, including leisure activities such as playing Ma-jong, chess, or cards, and social activities such as interacting with friends or connecting with the community, including being engaged in community-related organizations or clubs; (ii) self-improvement, including activities such as going to a social club, a sporting club, or another type of club, as well as taking a class or attending a training program; and (iii) prosocial behavior, including doing organized voluntary or charitable work and personally providing help to close people living separated from them, as well as sick or disabled strangers in need. The measurement of these three composite indices was also based on selecting the highest frequency of activities.

#### 2.2.3. Control Variables

Demographic information for WVS included gender (0 = “Female”, 1 = “Male”), age, educational attainment, residence (0 = “Village”, 1 = “The center of a city or town”), and marital status (0 = “Unmarried “, 1 = “Married or domestic partnership”). As the CHARLS measured ethnicity (0 = “Ethnic minorities”, 1 = “Han”), it was added in the analysis to better control for demographic variables.

### 2.3. Statistical Analysis

Firstly, the descriptive statistics were calculated for the variables in this study. Then, we conducted correlation and regression analyses to examine the relationships between the main variables. To examine the role of internet use in health outcomes, we utilized hierarchical linear regression models. SPSS (version 24; IBM Corp) was used for data analysis.

## 3. Results

### 3.1. Demographics

Table 1 and Table 2 present the participants’ details for Sample 1 and Sample 2, respectively. Sample 1 (N = 598; 283 (47.3%) males) had an average age of 64.76 (*SD* = 2.97), of which 330 (55.2%) were urban dwellers and 95 (15.9%) were unmarried. Of the participants, 343 (57.4%) were internet users.

The average age of Sample 2 (N = 9434; 4717 (50.0%) males) was 68.53 (*SD* = 6.44), of which 2526 (26.8%) lived in urban areas and 1855 (19.7%) were unmarried. The majority of participants identified as Han (N = 8774, 93.0%), and 7.0% as other ethnic minorities. The difference in residence and educational attainment between participants in the two samples might be related to different sampling methods and response rates. As urban district or rural area was included in the stratification standards of the CHARLS study, the response rate was higher in rural areas (91.4%) than in urban areas (74.6%) [46].

Of the participants, 628 (6.7%) had used the internet in the last month. Of those internet users, 577 (91.9%) reported chatting or news-watching behaviors, and the rest engaged in activities such as watching videos. Consequently, it is reasonable to consider internet use as a type of social engagement.

Regarding social engagement, 4779 (50.7%) of the total sample reported no participation in any of the listed common social activities in the previous month, while 1194 (12.7%) exhibited prosocial behavior. Entertainment (N = 3842, 40.7%) was the most popular activity among older people, and self-improvement (N = 568, 6.0%) was the least popular.

### 3.2. Correlation Analysis

Internet use was found to be positively correlated with self-reported health in Sample 1 (*r* = 0.17, *p* < 0.001). Table 3 shows the relationships between the variables in Sample 2. There were significant negative correlations between social activities and the CES-D 10 total score (*r*s < −0.05, *p*s < 0.001) and significant positive correlations between social activities and self-reported health (*r*s > 0.04, *p*s < 0.001), with the relationships between internet use and both self-reported health (*r* = 0.09, *p* < 0.001) and depression (*r* = −0.14, *p* < 0.001) being strongest.

### 3.3. Regression Analysis

Table 4 shows the regression results of Sample 1. All of the demographic variables were included at Step 1 of the analysis, including gender, age, residence, educational attainment, and marital status. Educational attainment positively predicted the health status of participants (β = 0.12, *p* = 0.01), whereas the other variables had no significant predictive effect on the health status. Individuals’ education could no longer predict health status (β = 0.07, *p* = 0.11) after the inclusion of internet use at Step 2, which was able to positively predict their self-reported health (β = 0.16, *p* < 0.001). The variance in self-reported health explained by internet use was 2%.

Another two hierarchical regression analyses were conducted to examine the associations between social activities and depression (Table 5) and self-reported health (Table 6) in Sample 2. First, demographic variables were included at Step 1, and three different social activity categories were included at Step 2, and internet use at Step 3.

Compared with females, males were less depressed (β = −0.15, *p* < 0.001) and they also reported greater health status (β = 0.005, *p* < 0.001). People living in the center of a city or town had lower depression scores (β = −0.13, *p* < 0.001) and higher health scores (β = 0.06, *p* < 0.001) than those living in villages. Marriage or domestic partnership also helped reduce depression (β = −0.07, *p* < 0.001). In addition, younger or better-educated participants were less depressed (βs > −0.07, *p*s < 0.001) and healthier (βs > −0.04, *p*s < 0.001).

After controlling for demographic variables, the frequency of entertainment, self-improvement, and prosocial behavior could significantly predict two health indicators. More specifically, more participation in activities such as exercising and studying (β = 0.05, *p* < 0.001), interacting with friends (β = 0.03, *p* = 0.01), and doing organized voluntary work (β = 0.03, *p* = 0.001) indicated better health status, as well as less depression (βs < −0.02, *p*s < 0.05).

More importantly, after controlling for the variables above, internet use still negatively predicted depression (β = −0.05, *p* < 0.001) and positively predicted health status (β = 0.04, *p* < 0.001). The variances in depression and self-reported health explained by internet use were both 0.2%.

## 4. Discussion

The present research was designed to investigate the association between internet use and the health of older Chinese adults through nationally representative samples. According to the results of two samples, internet use was a positive factor for health outcomes among Chinese older adults, specifically as internet use positively predicted self-reported health and negatively predicted depression. In particular, we examined the role of internet use as a new form of social engagement and compared the online approach to traditional offline social engagement activities in Sample 2. The results based on Sample 2 revealed that over half of the older adults from China reported participation in social activities, and they considered internet use as one of the approaches to meeting social needs, as more than 90% of those internet users reported chatting or news-watching behaviors.

Our study is the first to reveal the benefits of online social engagement and compare it with traditional types. Previous findings suggested that the number of older people browsing health care information through the internet is increasing [47,48]. Given this, it is not surprising that internet use has been shown to benefit self-treatment of common ailments and affect older people’s medical decisions [49]. However, it was the value of the internet for “social connection” rather than as an “information channel” that was the focus of this study, which was in line with our findings that older adults generally use the internet for chatting or watching news rather than obtaining health information. After accounting for demographic variables and the frequency of traditional social activities (e.g., entertainment, self-improvement, and prosocial behavior), our findings provided evidence for the association of internet use with both higher self-reported health and lower depressive symptom scores. The findings show that online socializing is as effective as traditional forms of socializing for improving health outcomes in older adults.

The growing trend for engaging in home-bound and solitary leisure activities is inevitable in older adults [13], and examining the relationships of these activities with health and exploring the most beneficial activities are essential for active aging. Older adults’ internet use was positively related to the different dimensions of subjective well-being [50] and health [51]. Furthermore, a longitudinal study found that the proportion of older Chinese adults using the internet for their social functions increased from 1.5% to 7.6% between 2014 and 2018, with the levels of life satisfaction improving [52]. Moreover, using the internet has been longitudinally linked to the preservation of individual cognitive functioning as people become older [53].

Recent studies also showed that social engagement could benefit older people, and the numerous functions of social engagement as an effective buffering resource in dealing with the stress process of illness and physical impairment of people in old age have been verified [12]. Men’s involvement in social activities, such as attending classes or lectures, reduced the mortality risk during the 9- to 12-year follow-up period [54]. Kobayashi et al. [23] found that participation in cultural activities, such as attending the cinema, theater, art galleries and museums, promoted the maintenance of functional literacy skills in older adults. In addition, older people who volunteer experience fewer depressive symptoms [37,41] and have lower mortality rates than those who do not [55]. In line with prior research, our results indicated that attending entertainment activities (e.g., interacting with friends, playing Ma-jong), self-improvement activities (e.g., attending a training course), and prosocial behavior (e.g., volunteering, providing help to family) all negatively predicted depressive symptom scores and positively predicted self-reported health scores after we controlled for participants’ gender, age, residence, education, marital status, and ethnicity.

Unexpectedly, the association between prosocial behavior and depressive symptom scores was not significant after internet use was included in the model, whereas self-improvement and entertainment activities negatively predicted depression. the other two offline activities. One explanation is that although we distinguished different activities from each other and examined the relationships separately, the behaviors themselves were not isolated but co-occurred [56].

Additionally, consistent with previous studies [57,58,59], the results of Sample 2 indicated that the health of females and the older participants were poorer, as reflected by both health indices. Moreover, living in the center of a city or town was found to be connected with better health outcomes, which could be attributed to abundant health facilities and human resources in economically developed cities [60]. Education could be another protective factor for depression, with highly educated individuals having lower depressive symptom scores and higher self-rated health scores. Compared with the unmarried, people who have married have a lower risk of depression. However, no difference was found between the health status of married and unmarried people. These results indicated that intimacy is more crucial to mental health than physical health among older adults. In relation to ethnicity, we found no effect of ethnicity on depression and health status, meaning that there was no difference in depression and health status between Han Chinese and ethnic minorities.

The analysis of Sample 1 provided less significant results than that of Sample 2. Specifically, education was the only demographic variable that significantly predicted participants’ self-rated health as in Sample 2, and the predictive effect was limited in Model 1. In other words, none of the factors of gender, age, residence, or marital status showed a predictive effect on health in Sample 1. As to the reasons for the difference in results (Table 4 vs. Table 6), the sample size might be an important factor as there were many more participants in Sample 2 (N = 9434) than in Sample 1 (N = 598). In addition, the discrepancy in demographic information, such as age distribution, influenced by data cleaning and different sampling methods, could not be ignored.

Therefore, our study verified the effectiveness of internet use in maintaining health during aging, showing that physical contact with others is not the only way to meet social needs. And chatting or watching news online can be useful alternatives to realize social engagement and reduce the risk of depression if physical contact is limited by environmental conditions.

In terms of implications for active aging, first, the Chinese government could help create a better network environment to suit the social needs of older adults. This is particularly important in the context of COVID-19, which has profoundly influenced their mental health, social engagement, and physical activity [61]. Second, considering the finding that internet access decreased the gap between urban and rural hypertension awareness and treatment [24], there is a need to develop a systematic internet-based health platform for seeking health information, monitoring physical health and making medical decisions.

Two nationally representative samples were used in our study. However, our study still suffered from some limitations. First, this cross-sectional study lacks experimental effects. It is possible that people who are not suffering from depression or other diseases have a better health status both physically and mentally, which allows them to participate in social activities.

Secondly, although the two datasets in our study are both widely used, the size of the target group in the WVS dataset and the predictive effect of internet use shown in the regression models in Sample 2 were both limited. The results were thought to be meaningful mainly because the analysis of both samples verified the predictive role of internet use on health. In addition, despite the fact that the number of older internet users has increased year over year, they only account for 11.5% of this age group [52]. Given the small sample size of older internet users, the small predictive effect was both acceptable and critical. However, our results still should be verified in future studies using an experimental design or longitudinal studies, and more potential covariates should be included to provide reliable causal conclusions. Moreover, further studies are required to discover the underlying mechanisms of internet use on older adults’ health, to improve user experience in response to social demand, and to promote active aging policies.

Another limitation is the lack of timeliness, as the surveys were both conducted in 2018, before the outbreak of COVID-19. Thus, this research cannot be said to directly improve our understanding of the actual impact of social activities, especially internet use, on older adults’ health during this special period. However, many studies, such as a systematic review [62] and empirical studies in China [63] and Japan [64], have suggested that lockdown in countries has caused an increase in internet use, with a growth rate of approximately 52% [65]. This was not only because the use of the internet for entertainment replaced outings during lockdown [64], but also because people needed to cope with the anxiety triggered by the pandemic and turned to the internet and social media [66]. On the one hand, these findings indicate a rise in internet use during COVID-19. On the other hand, our research shows that among all the social activities, internet use was not the most common, as no more than 10% of participants reported internet usage within the previous month. Despite the modest number of internet users in this study, substantial relationships were discovered between the online form of social engagement and both depression and self-rated health. Hence, although comparing pre-pandemic and post-pandemic findings requires additional research, we can reasonably predict that our results would be supported and the predictive effect would be larger.

## 5. Conclusions

The health of older adults has been a hot topic of academic research. Exploring the health status and its potential influencing factors among older adults can help us identify preventive measures that protect the physical and mental health of older people. According to this study, internet use reduces the chance of older adults experiencing depressive symptoms and improves their mental health. This research contributes to the existing body of research on the favorable link between social engagement and health outcomes among Chinese older adults with further examination of the association between the social effect of internet use and older adults’ health.

## Figures and Tables

**Table 1:** Descriptions of the study participants in Sample 1 (N = 598).

Variable		Count or Mean	Percentage (%)
Gender			
	Male	283	47.3
	Female	315	52.7
Age, mean (SD)		64.76 (2.97)	
Residence			
	Center of a city or town	330	55.2
	Village	268	44.8
Education			
	Primary school or below	322	53.8
	Middle school	158	26.4
	High school or vocational school	93	15.6
	Bachelor degree	23	3.8
	Master degree or above	2	0.3
Marital status			
	Married or domestic partnership	503	84.1
	Unmarried	95	15.9
Internet use, mean (SD)		3.03 (1.87)	

**Table 2:** Descriptions of the study participants in Sample 2 (N = 9434).

Variable		Count or Mean	Percentage (%)
Gender			
	Male	4717	50.0
	Female	4717	50.0
Age, mean (SD)		68.53 (6.44)	
Residence			
	Center of a city or town	2526	26.8
	Village	6908	73.2
Education			
	Primary school or below	6957	73.7
	Middle school	1541	16.3
	High school or vocational school	803	8.5
	Associate degree	70	0.7
	Bachelor degree	60	0.6
	Master degree or above	3	0.03
Marital status			
	Married or domestic partnership	7579	80.3
	Unmarried	1855	19.7
Ethnicity			
	Han	8774	93.0
	Ethnic minorities	660	7.0
Entertainment, mean (SD)		0.87 (1.19)	
Self-improvement, mean (SD)		0.15 (0.63)	
Prosocial behavior, mean (SD)		0.18 (0.54)	
Internet use, mean (SD)		0.19 (0.71)	

**Table 3:** Correlation analysis of variables in Sample 2 (N = 9434).

		1	2	3	4	5	6
1	Entertainment	1					
2	Self-improvement	0.12 ***	1				
3	Prosocial behavior	0.17 ***	0.10 ***	1			
4	Internet use	0.10 ***	0.15 ***	0.12 ***	1		
5	Depression	−0.07 ***	−0.08 ***	−0.05 ***	−0.14 ***	1	
6	Self-reported health	0.04 ***	0.07 ***	0.05 ***	0.09 ***	−0.36 ***	1

***, *p* < 0.001.

**Table 4:** Hierarchical regression analysis on health status in Sample 1 (N = 598).

		Model 1		Model 2	
		β	*p*	β	*p*
Step 1	Gender	0.00	0.93	0.00	0.92
	Age	0.05	0.21	0.06	0.12
	Residence	−0.01	0.90	−0.03	0.50
	Education	0.12	0.01	0.07	0.11
	Marital status	0.05	0.29	0.04	0.39
Step 2	Internet use			0.16	<0.001
*R* ^2^		0.019	0.049	0.039	<0.001
Δ*R*^2^		0.019	0.049	0.020	<0.001

**Table 5:** Hierarchical regression analysis on depression in Sample 2 (N = 9434).

		**Model 1**		**Model 2**		**Model 3**	
		**β**	***p***	**β**	***p***	**β**	***p***
Step 1	Gender	−0.15	<0.001	−0.16	<0.001	−0.16	<0.001
	Age	0.05	<0.001	0.04	<0.001	0.04	<0.001
	Residence	−0.13	<0.001	−0.12	<0.001	−0.11	<0.001
	Education	−0.12	<0.001	−0.11	<0.001	−0.10	<0.001
	Marital status	−0.07	<0.001	−0.08	<0.001	−0.08	<0.001
	Ethnicity	−0.02	0.07	−0.02	0.10	−0.02	0.10
Step 2	Entertainment			−0.06	<0.001	−0.06	<0.001
	Self-improvement			−0.05	<0.001	−0.05	<0.001
	Prosocial behavior			−0.02	0.03	−0.02	0.06
Step 3	Internet use					−0.05	<0.001
*R* ^2^		0.085	<0.001	0.092	<0.001	0.094	<0.001
Δ*R*^2^		0.085	<0.001	0.008	<0.001	0.002	<0.001

**Table 6:** Hierarchical regression analysis on health status in Sample 2 (N = 9434).

		**Model 1**		**Model 2**		**Model 3**	
		**β**	***p***	**β**	***p***	**β**	***p***
Step 1	Gender	0.05	<0.001	0.06	<0.001	0.06	<0.001
	Age	−0.04	<0.001	−0.04	<0.001	−0.04	<0.001
	Residence	0.06	<0.001	0.05	<0.001	0.04	<0.001
	Education	0.05	<0.001	0.04	<0.001	0.03	0.02
	Marital status	0.02	0.14	0.02	0.13	0.02	0.14
	Ethnicity	0.02	0.05	0.02	0.06	0.02	0.06
Step 2	Entertainment			0.03	0.01	0.02	0.02
	Self-improvement			0.05	<0.001	0.05	<0.001
	Prosocial behavior			0.03	0.001	0.03	0.003
Step 3	Internet use					0.04	<0.001
*R* ^2^		0.014	<0.001	0.020	<0.001	0.021	<0.001
Δ*R*^2^		0.014	<0.001	0.005	<0.001	0.002	<0.001

## Data Availability

The datasets used in this research are available on reasonable request from the corresponding author.
